# Serum Proteinogram in the Free‐Living Brazilian Common Opossum (*Didelphis aurita*)

**DOI:** 10.1002/elps.70016

**Published:** 2025-08-23

**Authors:** Andrés Mauricio Ortega Orozco, Lucas Drumond Bento, Pollyanna Cordeiro Souto, Fabrícia Modolo Girardi, Verônica Rodrigues Castro, João Vitor Gonçalves de Oliveira, Camilo José Ramirez Lopez, Edvaldo Barros, Artur Kanadani Campos, Leandro Abreu da Fonseca

**Affiliations:** ^1^ Veterinary Department Universidade Federal de Viçosa Viçosa Minas Gerais Brazil; ^2^ Biomolecule Analysis Center (NuBioMol) Universidade Federal de Viçosa Viçosa Minas Gerais Brazil; ^3^ Veterinary Clinical Pathology Laboratory at Veterinary Department Universidade Federal de Viçosa Viçosa Minas Gerais Brazil

**Keywords:** LC–MS, proteomic, sodium dodecyl sulfate‐polyacrylamide gel electrophoresis (SDS‐PAGE), wildlife

## Abstract

*Didelphis aurita* is a synanthropic marsupial widely distributed in southeastern Brazil, known for its resistance to venom and its relevance in biomedical research. This study aimed to characterize the serum proteinogram of free‐living *D. aurita* individuals. Blood samples from 27 animals, classified as “healthy” or “diseased,” were analyzed. Eighteen protein bands were identified, with molecular weights ranging from 24 to 242 kDa. Among these, variations in specific bands were associated with health status (band J), sex (bands D, M, N, and P), and age (bands N and P). Mass spectrometry (liquid chromatography–tandem mass spectrometry [LC–MS/MS]) identified seven proteins, including DM64, ceruloplasmin, von Willebrand factor A (VWFA) domain protein, alpha‐2‐macroglobulin, fibronectin, and actin depolymerizing factor. These results highlight the influence of biological factors on serum protein profiles and reinforce the potential of *D. aurita* as a model for immunological and proteomic studies.

## 1 Introduction

The Brazilian common opossum (*Didelphis aurita*) is a marsupial species belonging to the genus Didelphis (Linnaeus, 1758). This genus comprises six endemic species distributed across the American continent, with five occurring in South America and four present in Brazil [1]. The Brazilian representatives include *Didelphis albiventris*, *Didelphis imperfecta*, *Didelphis marsupialis*, and *D. aurita* [2].

*D. aurita* inhabits primarily eastern Brazil, ranging from Alagoas to Santa Catarina states, with distribution extending to Mato Grosso do Sul, southeastern Paraguay, and the Misiones Province in Argentina [2, 3]. This medium‐sized marsupial exhibits a body mass of 900–3000 g [4] and displays characteristic frugivorous/omnivorous feeding behavior, playing a significant ecological role as a seed disperser in neotropical ecosystems [5, 6].

Opossums are widely recognized as synanthropic animals, commonly found in urban and peri‐urban environments [7, 8]. Their close proximity to human populations has led to diverse interactions, including their use as a protein source through bushmeat consumption and as components of traditional medicine in certain communities [9, 10]. Beyond their cultural and subsistence roles, opossums are of considerable scientific interest due to their dual biomedical significance. On one hand, their serum contains proteins capable of neutralizing snake venom, which have been the subject of extensive research for potential therapeutic applications in human medicine [11, 12, 13]. On the other hand, their role as reservoirs of zoonotic pathogens positions them as key species in the context of One Health approaches to disease surveillance and prevention [7].

However, Brazilian marsupials function as reservoirs for numerous pathogenic agents with documented zoonotic potential [14]. Didelphid species demonstrate susceptibility to diverse pathogens, including bacteria [15], protozoans [8, 16], viruses [17], and nematodes [18], all of which constitute significant threats to both domestic animal and human health.

Serum protein electrophoresis using sodium dodecyl sulfate‐polyacrylamide gel electrophoresis (SDS‐PAGE) [19] enables molecular weight‐based protein separation [20]. When combined with liquid chromatography–tandem mass spectrometry (LC–MS/MS), this approach significantly enhances protein identification sensitivity. These macromolecules perform diverse biological functions, particularly acute‐phase proteins (APPs) that exhibit concentration fluctuations in response to inflammatory stimuli and serve as valuable biomarkers across animal species [21].

Proteomic approaches enable the identification of serum proteins that may serve as diagnostic biomarkers or monitoring tools for these pathological conditions. Therefore, this study aims to characterize the serum protein profile of free‐ranging Brazilian common opossum (*D. aurita*) in Viçosa, Minas Gerais, Brazil.

## 2 Materials and Methods

### 2.1 Data Collection

This study was approved by the Ethics Committee for the Use of Animals (CEUA/UFV) under protocol number 30/2021 and was authorized by the Biodiversity Information and Authorization System (SISBIO) of the Brazilian Institute of Environment and Renewable Natural Resources (IBAMA) under license number 64930‐3.

The animals used in the study were captured in the municipality of Viçosa, located in the state of Minas Gerais, Brazil (22°45′14″ S; 42°52′55″ W). The region spans an area of 299.418 km^2^ and has a humid subtropical climate, classified as Cwa according to the Köppen‐Geiger system, with an average annual temperature of 20.4°C and an average annual rainfall of 1.250 mm (https://en.climate‐data.org/south‐america/brazil/minas‐gerais/vicosa‐25021/).

Each animal was identified and categorized by sex, age, and age group according to the criteria established by Macedo et al. [22]. They were further classified as either “healthy” or “diseased” on the basis of findings from physical examinations and/or additional diagnostic tests that indicated possible injuries or infectious agents.

Captures took place between March and July 2021 using Tomahawk traps (0.45 × 0.21 × 0.21 m^3^) baited with a mixture of sardines, cornmeal, and banana.

Animals were physically restrained for sample collection. Blood was drawn via venipuncture of the lateral coccygeal vein, following the method described by Carvalho do Nascimento and Horta [4]. Samples were collected with 13.0 × 4.5 mm^2^ disposable needles attached to 5 mL syringes and transferred to 4 mL clot activator tubes (Becton and Dickinson Co., Franklin Lakes, NJ, USA) for biochemical and electrophoretic analysis. Serum was separated by centrifugation at 3000 rpm for 5 min and stored at −20°C. Additional samples were collected in 4 mL EDTA tubes (tripotassium) for cell counts, and blood smears were prepared and stained using Romanowsky stains to detect hemoparasites.

### 2.2 Protein Analysis

Total serum protein concentrations were measured using the biuret method with commercially available reagents. Absorbance readings were taken on an automated spectrophotometer at the Clinical Laboratory of the Veterinary Department at the Federal University of Viçosa.

Serum proteins were separated by SDS‐PAGE, following a modified version of the method described by Laemmli [19]. The procedure was performed using a vertical electrophoresis system (PROTEAN II XI Vertical Electrophoresis Cells, Bio‐Rad). Gels containing 20 wells were prepared, with each well loaded with 30 µL of PBS buffer, 20 µL of sample buffer, and 10 µL of serum. Electrophoresis was run initially at 100 V for 1 h, then increased to 150 V, and finally to 200 V until completion.

After electrophoresis, the gels were stained with Coomassie brilliant blue R‐250, then left overnight in a destaining solution, and later stored in a 5% acetic acid solution. The resulting protein bands were scanned in transparency mode at 300 dpi using an ImageScanner III (GE Healthcare, Sweden). The scanned images were saved in both .tif and .png formats. Gels were preserved in 5% acetic acid until the enzymatic digestion step.

The molecular weights and relative concentrations of protein fractions were determined via computerized densitometry using ImageLab software (Loccus, 2017), on the basis of the scanned gel images. Molecular weight markers ranging from 10 to 250 kDa (Precision Plus Protein Standards, Bio‐Rad) were used to estimate band sizes, and standard curves were generated from these markers to guide the densitometric analysis.

Protein bands obtained from one‐dimensional (1D) SDS‐PAGE were excised, finely cut, and subjected to enzymatic digestion, following the methodology described by Shevchenko et al. [23]. The gel fragments were transferred to 1.5 mL microtubes containing 200 µL of 50% (v/v) acetonitrile in 25 mM ammonium bicarbonate buffer (pH 8.0) and washed to remove residual dye and SDS. Dehydration was performed by adding 200 µL of 100% acetonitrile twice, each with a 5‐min incubation. The fragments were then dried using a vacuum centrifuge (model AG‐22331, Eppendorf, Hamburg, Germany) for 15 min.

For protein reduction, fragments were incubated in 100 µL of 65 mM dithiothreitol (DTT) in 100 mM ammonium bicarbonate buffer (pH 8.0) at 56°C for 30 min. Subsequently, alkylation was carried out using 100 µL of 200 mM iodoacetamide in the same buffer at room temperature for 30 min in the dark. Following this, the fragments underwent two cycles of rehydration and dehydration using ammonium bicarbonate and acetonitrile, respectively. Finally, the gel fragments were once again dried in a vacuum centrifuge under the previously described conditions.

During the proteolytic digestion phase, gel fragments were rehydrated on ice with 100 µL of a porcine pancreas trypsin solution (proteomics grade, TPCK‐treated; reference V5111, Promega Corporation, Wisconsin, USA), prepared at a final concentration of 25 ng/µL in an activation buffer composed of 40 mM ammonium bicarbonate (pH 8.0) and 10% acetonitrile. Following a 45‐min incubation on ice, an additional 130 µL of the activation buffer was added to each tube containing the gel fragments.

Samples were then incubated in a water bath at 37°C for 22 h to complete enzymatic digestion. After proteolysis, the tubes were sonicated for 10 min and briefly vortexed for 20 s. The resulting supernatant was carefully transferred to a clean microtube.

To extract remaining peptides, 150 µL of a solution containing 5% (v/v) formic acid in 50% (v/v) acetonitrile was added to the remaining gel pieces. Tubes were vortexed for 20 s, held at room temperature for 15 min, and then sonicated for an additional 2 min. The resulting solution was collected and combined with the previously transferred supernatant. This extraction step was repeated once more, and the combined extracts were pooled into the same microtube. Peptide‐containing samples were then concentrated using a vacuum centrifuge and stored at −20°C until mass spectrometry analysis.

Peptide replicates obtained from the 1D SDS‐PAGE bands were solubilized in 80 µL of 0.1% aqueous formic acid. Of this solution, 70 µL were transferred to appropriate vials and analyzed by LC–MS/MS using a nanoAcquity UPLC system (Waters, Milford, USA) coupled to an Amazon Ion Trap mass spectrometer (Bruker Daltonics, Bremen, Germany). Chromatographic separation was performed on a trap column followed by a C18 BEH130 capillary column (1.7 µm, 100 µm × 100 mm), operated at a flow rate of 0.400 µL/min.

Peptides were eluted automatically and introduced into the mass spectrometer in online mode via a nano‐electrospray ionization (nanoESI) source. The mobile phase consisted of (A) water with 0.1% (v/v) formic acid and (B) acetonitrile with 0.1% (v/v) formic acid. The gradient program began with a 5‐min desalting step at 5% B, followed by a linear increase to 50% B over 40 min, held at 50% B for 5 min. Then, B was ramped from 50% to 90% over 10 min, maintained at 90% for 5 min, and decreased back to 10% over 5 min, followed by a 5‐min hold at 10%.

Mass spectrometry data were acquired in positive ion mode. Full MS1 scans were collected over the *m*/*z* range of 300–1500, and MS2 scans over 70–3000 *m*/*z*. The data acquisition period lasted approximately 75 min. The Ion Trap instrument operated in auto‐MSn mode, automatically selecting and fragmenting the most intense precursor ions in each scan, excluding singly charged ions.

Data acquisition was controlled by Hystar software (version 3.2, Bruker Daltonics), and spectral processing was conducted using Data Analysis software (version 4.0, Bruker Daltonics) with default proteomics settings. Peak lists were exported in mzXML format using CompassXport (version 3.0, Bruker Daltonics).

### 2.3 Data Analysis

A two‐way ANOVA was performed to evaluate the effects of sex (male and female) and age group (adult and juvenile). Comparisons between healthy and sick animal groups were conducted using mean comparison tests. Data normality was assessed with the Shapiro–Wilk test. For normally distributed variables, parametric comparisons were made using Student's *t*‐test, as ANOVA factors with a single degree of freedom are statistically equivalent to the *F*‐test. For variables that did not meet the normality assumption, non‐parametric analyses were conducted using the Mann–Whitney *U* test. A significance level of 5% (*p* < 0.05) was applied to all statistical tests.

## 3 Results

A total of 27 animals were captured, comprising 17 females (63%, *n* = 27) and 10 males (37%, *n* = 27). Among these, 20 were adults (74%, *n* = 27) with a sex distribution of 7 males and 13 females, whereas the remaining 7 were juveniles (26%, *n* = 27) consisting of 4 males and 3 females. Eighteen captured individuals were classified as diseased (67%, *n* = 27). The associated comorbidities observed in these animals are detailed in Table 1.

**TABLE 1:** Pathological conditions detected in the physical or laboratory evaluation of captured *Didelphis aurita*.

ID	Sex	Age	Pathological condition
G 02	F	A	Hemoparasitosis
G 04	M	J	Neoplasms in the mouth and forelimbs
G 05	M	J	Hemoparasitosis
G 06	F	A	Ocular infection/Hemoparasitosis
G 10	M	A	Hemoparasitosis
G 12	F	A	Hemoparasitosis
G 13	F	A	Subcutaneous nodule on the left forelimb
G 14	F	A	Hemoparasitosis
G 15	F	J	Hemoparasitosis
G 16	F	A	Hemoparasitosis
G 17	M	A	Hemoparasitosis
G 18	F	A	Hemoparasitosis
G 19	F	A	Hemoparasitosis
G20	F	A	Hemoparasitosis
G 22	M	A	Hemoparasitosis
G 23	F	A	Hemoparasitosis
G 25	F	J	Hemoparasitosis
G 26	F	A	Jaundice

Abbreviations: A, adult; F, female; J, juvenile; M, male.

**FIGURE 1:**
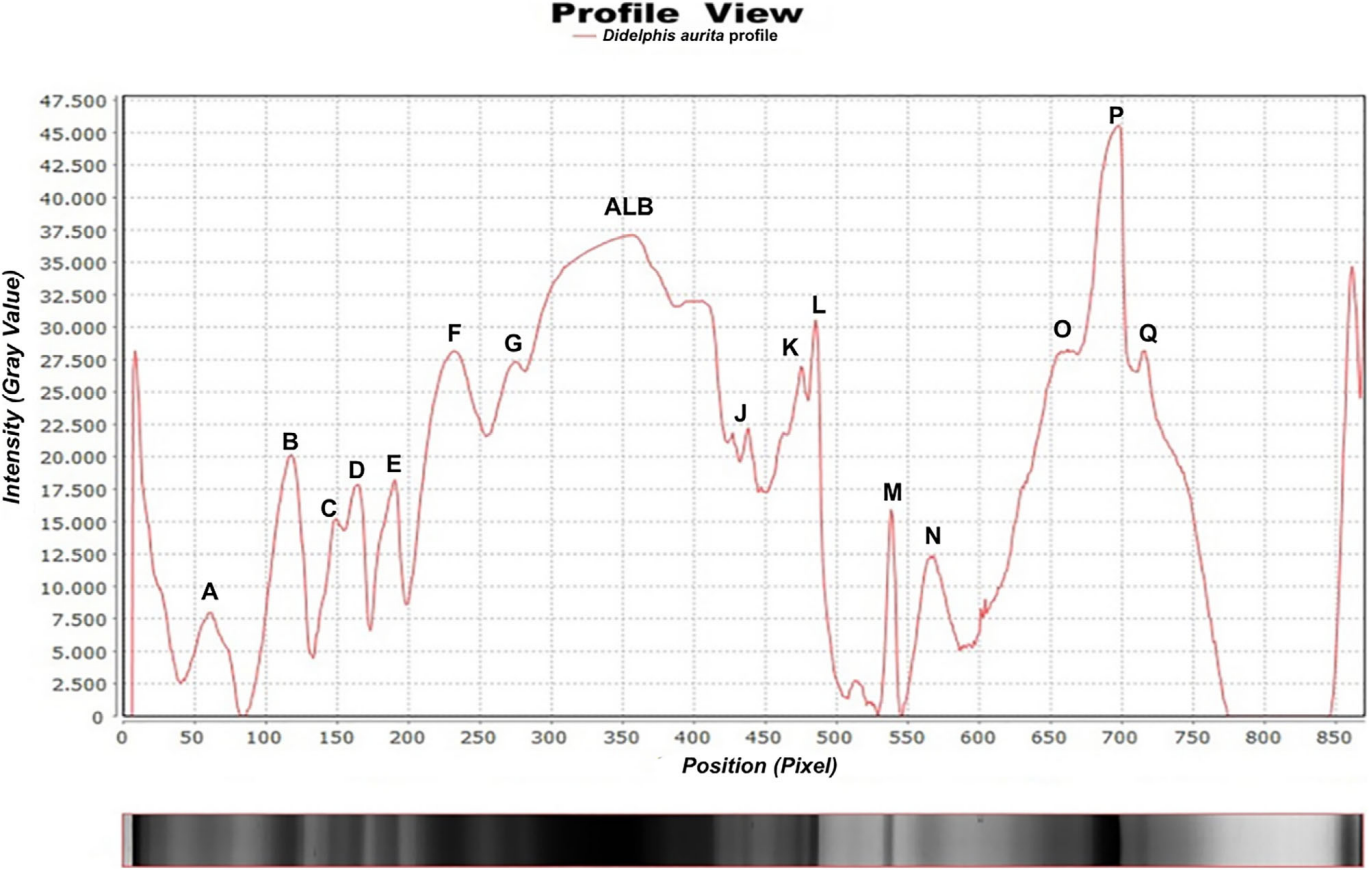
Electrophoretic profile of serum proteins from *Didelphis aurita*. Flagged bands. ALB, albumin. The different letters identify the respective peaks/bands after electrophoresis.

SDS‐PAGE electrophoresis identified 18 distinct protein bands (Figure 1), with quantitative results tabulated and expressed as mean ± standard deviation values (Table 2).

**TABLE 2:** Descriptive statistics of molecular weights and concentrations of different protein bands obtained by sodium dodecyl sulfate polyacrylamide gel electrophoresis (SDS‐PAGE) electrophoresis of *Didelphis aurita* serum.

Band	Molecular weight (kDa)	Concentration (mg)
Total protein		7268 ± 973
A	242 ± 7	328 ± 172
B	194 ± 19	374 ± 214
C	158 ± 17	179 ± 106
D	139 ± 15	165 ± 101
E	93 ± 4.6	283 ± 100
F	76 ± 2.5	1165 ± 501
G	74 ± 1.3	1457 ± 918
Albumin	66 ± 0.3	2932 ± 335
I	55 ± 3	668 ± 292
J	46 ± 3	800 ± 195
K	42 ± 2	217 ± 70
L	40 ± 0.9	251 ± 132
M	38 ± 0.7	261 ± 139
N	35 ± 0.4	65 ± 38
O	32 ± 1	289 ± 129
P	26 ± 0.6	1288 ± 627
Q	24 ± 0.3	1293 ± 233

Statistical analysis demonstrated that the concentration of specific serum protein bands was significantly influenced by intrinsic biological variables and health status (Table 3). Band N (35 kDa) exhibited significant variation associated with both sex and age, whereas bands D (139 kDa) and albumin (ALB) (66 kDa) were modulated by age. Band J (46 kDa) showed differential expression between clinically healthy and diseased individuals. These results underscore the impact of physiological and pathological factors on the serum proteomic profile of *D. aurita*, supporting its utility as a model in comparative proteomics.

**TABLE 3:** Influence of sex, age, and clinical status on serum protein band concentrations in *Didelphis aurita*.

Band/Protein	Molecular weight (kDa)	Sex effect (p)	Age effect (p)	Health status effect (p)
D	139 ± 15	Yes (0.0063)	No	No
ALB	66 ± 0.3	No	Yes (0.0218)	No
J	46 ± 3	No	No	Yes (0.0467)
N	35 ± 0.4	Yes (0.0109)	Yes (0.0263)	No
P	26 ± 0.6	Yes	Yes	Not significant

Abbreviation: ALB, albumin.

Through two‐way ANOVA, we determined that concentrations of proteins “D” (*p* = 0.0064) and “M” (38 ± 0.7 kDa; *p* = 0.031) were significantly influenced by animal sex. Protein “N” concentrations showed sex‐ and age‐dependent variations, with significant differences observed between adult females and adult males (*p* = 0.0058), as well as between adult females and juvenile females (*p* = 0.0013). Protein “P” (26 ± 0.6 kDa; *p* = 0.0353) demonstrated significant sex‐ and age‐related differences specifically between adult and juvenile females (*p* = 0.0105). Using LC–MS/MS technology, we achieved nominal identification of seven proteins, with their respective names and theoretical masses detailed in Table 4.

**TABLE 4:** Proteins identified by liquid chromatography–tandem mass spectrometry (LC–MS/MS) analysis.

Accession	Band	Name	MW theoretical (kDa)
Q8MIS3	B	Venom myotoxin inhibitor DM64	55.97
F6T3A4	C	Ceruloplasmin isoform X2	120.22
A0A5F8G303	C	VWFA domain‐containing protein	55.36
F7B8I0	D	Alpha‐2‐macroglobulin isoform X1	165.60
A0A5F8H7U7	F	Fibronectin	263.77
F7BJP5	H	Serum albumin	68.09
A0A5F8HGF5	O	Actin‐depolymerizing factor	81.43

Abbreviation: VWFA, von Willebrand factor A.

## 4 Discussion

Among the protein bands that could be identified, band “D” (139 ± 15 kDa) was classified as alpha‐2‐macroglobulin (A2M). This active functional protein (AFP), which belongs to the superfamily of proteins with a thioester domain, acts as a protease inhibitor and is involved in the immune response. This protein participates in multiple biological processes, such as the proliferation of T lymphocytes, the regulation of apoptosis, and the activation of macrophages [24, 25]. A2M, like other macroglobulins, has been recognized for its anti‐hemorrhagic properties, particularly in neutralizing snake venom metalloproteinases. This function has been observed in hedgehogs (*Erinaceus europaeus*) [26] and murine models [27]. In a study conducted by Ochoa et al. [28], through proteomic analyses, proteins present in the blood serum of the California ground squirrel (*Otospermophilus beecheyi*) were identified that interact with venom components, among which α‐2‐macroglobulin stood out. On the basis of these findings, it is plausible to suggest that A2M contributes to the natural resistance of didelphis to snake venom, and the A2M identified in *D. aurita* may serve a similar protective role.

In addition, A2M concentrations were influenced by age, consistent with previous reports in domestic species such as pigs, where APP levels tend to increase with age. This variation is likely due to the ongoing development in younger animals, resulting in lower concentrations of certain proteins until physiological maturity is reached [29]. The trend observed in *D. aurita* suggests a comparable pattern.

Additionally, band “J” (46 ± 3 kDa) differed significantly between the “healthy” and “sick” groups. Although its identity could not be definitively confirmed, its molecular weight aligns with that of haptoglobin (HP), which has a theoretical mass of 45.9 kDa in *Monodelphis domestica* (UniProt: F7BVM7). HP is an acute phase glycoprotein that plays a fundamental role in neutralizing free hemoglobin, preventing its toxic effects, and protecting tissues from oxidative damage. In addition, it acts as a natural antagonist in the activation of ligand receptors in the immune system [30, 31]. It also exhibits antimicrobial properties [32]. This protein is found in the plasma and serum of adults at reference concentrations ranging from 0.3 to 2 mg/mL [33]. Its levels tend to rise in situations involving inflammation, trauma, or burns, as well as in cases of various neoplasms. Reduced HP concentrations are observed in cases of hemolysis, malnutrition, ineffective erythropoiesis, hepatocellular disorders, and during late pregnancy [34, 35]. Interestingly, in this study, HP levels appeared to decrease in sick animals. As only one animal presented with icteric mucous membranes and no clear signs of liver disease were observed, further investigation using more sensitive techniques is recommended to confirm the identity of band “J” and clarify its role in health assessments of *D. aurita*.

The concentrations of proteins “M” and “N” (both with a molecular mass of 38 ± 0.7 kDa) were influenced by the sex of the individuals. It is well established that sex hormones modulate the inflammatory response. Kahl and Elsasser [36] demonstrated a significant increase in TNF‐α and APP levels in bulls following exogenous testosterone administration and LPS inoculation. Similarly, Bosanquet et al. [37] reported a pronounced acute phase response in male rats, marked by dramatic increases in alpha‐1‐macroglobulin (A1M) and A2M, with A2M levels rising up to 86‐fold. In contrast, female rats exhibited only minor or no changes. Coe and Ross [38] identified a “female protein” in females—an APP homologous to C‐reactive protein (CRP)—that naturally occurs at levels 5–10 times higher than in males. Interestingly, castrated males exhibited levels comparable to females, further underscoring the regulatory role of sex hormones. These findings support the idea that the expression profile of certain APPs may vary by sex, likely driven by hormonal differences. However, the underlying physiological and molecular mechanisms responsible for this modulation remain to be fully elucidated.

Similarly, protein “P” (26 ± 0.6 kDa) showed significant variation influenced by both sex and age (*p* < 0.05). Christoffersen et al. [39] found a similar pattern in minipigs, where inflammatory responses varied with sex and age, possibly due to differences in sex hormone concentrations associated with sexual maturity.

In this study, seven proteins were identified by LC–MS/MS, including fibronectin (FN), a multifunctional protein involved in cell adhesion [40], cell morphology regulation [41], and the acute phase response [42]. FN expression is modulated by pro‐inflammatory cytokines such as IL‐1 and IL‐6 [43]. Although FN is recognized as an acute phase protein in rodent models, similar behavior has not been consistently observed in humans [42].

Ceruloplasmin (CP), another protein identified, functions primarily as a copper transporter in plasma and also exhibits antioxidant properties [21, 42]. Changes in CP levels are related to hyperferritinemia and hepatic iron accumulation, contributing to the progression of more advanced liver fibrosis [44, 45]. Data on CP in marsupials remain scarce, but Thomas et al. [46] confirmed its enzymatic activity in koalas (*Phascolarctos cinereus*), suggesting its potential as an indicator of nutritional status in these animals.

Actin depolymerizing factor, identified in this study as “O” band, is a highly conserved protein found in eukaryotic cells, where it plays a key role in cytoskeletal remodeling. It functions by binding to actin filaments and promoting their rapid disassembly, thereby facilitating essential cellular processes such as locomotion, cytokinesis, and other morphodynamic activities [47, 48]. Actin dynamics is particularly important for *Apicomplexa* protozoa, which use a mechanism known as gliding motility to invade host cells. This process relies on continuous actin turnover, for which actin depolymerizing factor is essential [49, 50]. Although direct evidence is lacking, it is reasonable to hypothesize that Hepatozoon species may use a similar mechanism for erythrocyte invasion in *D. aurita*, possibly involving the same protein.

ALB, also detected in this study, is the most abundant plasma protein and plays a central role in the transport of endogenous and exogenous substances, as well as in maintaining oncotic pressure [21]. ALB levels typically decrease during inflammation, classifying it as a negative acute phase protein [21, 42].

Opossums are known for their natural resistance to snake venom, a trait associated with several serum proteins with anti‐hemorrhagic and anti‐myotoxic properties [12, 23, 51, 52]. One such protein is DM64, a glycoprotein found in *Didelphis* spp. and known to inhibit phospholipase A2 enzymes, which are major components of venom‐induced myotoxicity [52, 53]. Opossums synthesize glycoproteins like DM43 and oprin that counteract snake venom metalloproteinases by forming non‐covalent complexes, effectively neutralizing their toxic effects. Notably, they do not inhibit venom serine proteinases or metalloproteinases of bacterial origin, suggesting a high specificity for components unique to snake venom [54, 55, 56, 57]. Therefore, in this study was identified a venom myotoxin inhibitor DM64.

This study also identified proteins containing von Willebrand factor A (VWFA) domains, which are key structural motifs involved in protein–protein interactions. These domains are found in a wide variety of proteins, including von Willebrand factor (vWF), collagen types VI, VII, XII, and XIV, integrin subunits, and complement factors B and [58, 59, 60]. VWFA and VWFA‐like domains are also found in thrombospondin‐related proteins involved in parasite motility and host cell invasion via gliding motility—an essential mechanism used by apicomplexan parasites [61]. A study investigated the evolution of vWF in opossums, suggesting that it may have adapted to resist C‐type lectins (CTLs) present in snake venoms. In species from the Didelphini clade, vWF has shown signs of rapid evolution, with mutations that could prevent it from binding to CTLs. To test this hypothesis, the researchers analyzed the binding affinity between vWF variants and purified CTLs. The results revealed that resistant forms of vWF occur not only in Didelphini but also in other opossums of the Didelphidae family, indicating a possible widespread venom resistance in this group [57]. On the basis of these parallels, it is plausible that VWFA domain‐containing proteins also contribute to the ability of Hepatozoon to invade red blood cells in *D. aurita*, possibly through a mechanism similar to that of other apicomplexans.

Beyond the known implications for venom resistance and zoonotic surveillance under a One Health perspective, the identification of acute phase and venom‐neutralizing proteins in *D. aurita* highlights their potential as biomarkers for health status monitoring. These proteins may serve as valuable tools for future studies focused on population health assessments, environmental stress evaluations, and even rehabilitation strategies for injured or rescued animals. As such, this study provides not only foundational proteomic data but also opens avenues for applied research in wildlife health management.

## Conflicts of Interest

The authors declare no conflicts of interest.

## Data Availability

The authors have nothing to report.

## References

[1] G. Melo and J. Sponchiado , “Distribuição Geográfica dos Marsupiais no Brasil,” in Os Marsupiais Do Brasil: Biologia, Ecologia e Conservação UFMS, (2012): 93–100.

[2] R. Rossi , G. Bianconi , and P. W. Ordem Didelphimorphia , Mamíferos Do Brasil (Universidade Estadual de Londrina, 2006), 27–60.

[3] D. Astúa , N. De la Sancha , and L. Costa , IUCN Red List of Threatened Species: Didelphis Aurita IUCN Red List Threat. Species (2015), 10.2305/IUCN.UK.2015-2.RLTS.T40503A22176266.en.

[4] M. Passamani , “Análise da comunidade de marsupiais em Mata Atlântica de Santa Teresa, Espírito Santo,” Boletim do Museu de Biologia Mello Leitão 11 (2000): 215–228.

[5] N. C. Cáceres , “Diet of Three *Didelphid marsupials* (Mammalia, Didelphimorphia) in Southern Brazil,” Mammalian Biology 69 (2004): 430–433.

[6] N. C. Cáceres and E. L. A. Monteiro‐Filho , “Food Habits, Home Range and Activity of *Didelphis aurita* (Mammalia, Marsupialia) in a Forest Fragment of Southern Brazil,” Studies on Neotropical Fauna and Environment 36 (2001): 85–92.

[7] M. A. Bezerra‐Santos , R. A. N. Ramos , A. K. Campos , F. Dantas‐Torres , and D. Otranto , “Didelphis spp. Opossums and Their Parasites in the Americas: A One Health Perspective,” Parasitology Research 120 (2021): 4091–4111, 10.1007/s00436-021-07345-9. 33788021 PMC8599228

[8] A. K. M. Teodoro , A. A. Cutolo , G. Motoie , C. S. Meira‐Strejevitch , V. L. Pereira‐Chioccola , and T. M. F. Mendes , “Gastrointestinal, Skin and Blood Parasites in Didelphis spp. From Urban and Sylvatic Areas in São Paulo state,” Veterinary Parasitology: Regional Studies and Reports 16 (2019): 100286. 31027595 10.1016/j.vprsr.2019.100286

[9] I. O. Carneiro , N. J. Santos , N. S. Silva , et al., “Knowledge, Practice and Perception of Human‐Marsupial Interactions in Health Promotion,” Journal of Infection in Developing Countries 13 (2019): 342–347. 32045379 10.3855/jidc.10177

[10] F. B. Barros and P. de Aguiar Azevedo , “Common Opossum (*Didelphis marsupialis* Linnaeus, 1758): Food and Medicine for People in the Amazon,” Journal of Ethnobiology and Ethnomedicine 10 (2014): 65. 25209094 10.1186/1746-4269-10-65 PMC4167517

[11] I. R. León , A. G. da Costa Neves‐Ferreira , and S. L. G. da Rocha , et al., “Using Mass Spectrometry to Explore the Neglected Glycan Moieties of the Antiophidic Proteins DM43 and DM64,” Proteomics 12 (2012): 2753–2765. 22744933 10.1002/pmic.201200062

[12] V. A. Bastos , F. Gomes‐Neto , J. Perales , A. G. C. Neves‐Ferreira , and R. H. Valente , “Natural Inhibitors of Snake Venom Metalloendopeptidases: History and Current Challenges,” Toxins 8 (2016): 250, 10.3390/toxins8090250. 27571103 PMC5037476

[13] A. G. C. Neves‐Ferreira , J. Perales , J. W. Fox , et al., “Structural and Functional Analyses of DM43, a Snake Venom Metalloproteinase Inhibitor From *Didelphis marsupialis* Serum,” Journal of Biological Chemistry 277 (2002): 13129–13137. 11815628 10.1074/jbc.M200589200

[14] M. M. Bitencourt and A. M. R. Bezerra , “Infection Agents of Didelphidae (Didelphimorphia) of Brazil: An Underestimated Matter in Zoonoses Research,” Mammalia 86 (2022): 105–122.

[15] M. R. André , A. C. Calchi , L. Perles , et al., “Novel Ehrlichia and Hepatozoon Genotypes in White‐Eared Opossums (*Didelphis albiventris*) and Associated Ticks From Brazil,” Tick‐Borne Disease 13 (2022): 102022. 10.1016/j.ttbdis.2022.102022 35973262

[16] M. E. B. Santiago , R. O. Vasconcelos , K. R. Fattori , D. P. Munari , A. F. Michelin , and V. M. F. Lima , “An Investigation of Leishmania spp,” Veterinary Parasitology 150 (2007): 283–290. 17996372 10.1016/j.vetpar.2007.09.026

[17] F. L. P. Lavorente , A. de Matos , E. Lorenzetti , et al., “First Detection of Feline Morbillivirus Infection in White‐Eared Opossums (*Didelphis albiventris*, Lund, 1840), a Non‐Feline Host,” Transboundary and Emerging Diseases 69 (2022): 1426–1437. 33872470 10.1111/tbed.14109

[18] M. A. Bezerra‐Santos , L. F. V. Furtado , E. M. L. Rabelo , B. C. F. Nogueira , R. S. Yamatogi , and A. K. Campos , “High Prevalence of *Ancylostoma caninum* Infection in Black‐Eared Opossums (*Didelphis aurita*) in an Urban Environment,” Parasitology Research 119 (2020): 2343–2346. 32435896 10.1007/s00436-020-06708-1

[19] U. K. Laemmli , “Cleavage of Structural Proteins During the Assembly of the Head of Bacteriophage T4,” Nature 227 (1970): 680–685. 5432063 10.1038/227680a0

[20] P. D. Eckersall , “Proteins, Proteomics, and the Dysproteinemias,” in Clinical Biochemistry of Domestic Animals (Academic Press, 2008), 117–155.

[21] C. Cray , Acute Phase Proteins in Animals, In: PROGRESS in Molecular Biology and Translational Science. 1st ed. Amsterdam: Elsevier, 105 (2012): 113–150, 10.1016/B978-0-12-394596-9.00005-7. PMC7149966 22137431

[22] J. Macedo , D. Loretto , M. V. Vieira , and R. Cerqueira , “Classes De Desenvolvimento Em Marsupiais: Um Método Para Animais Vivos,” Mastozool Neotrop 13 (2006): 133–136.

[23] A. Shevchenko , H. Tomas , J. Havli , J. V. Olsen , and M. Mann , “In‐Gel Digestion for Mass Spectrometric Characterization of Proteins and Proteomes,” Nature Protocols 1 (2006): 2856–2860. 17406544 10.1038/nprot.2006.468

[24] J. Qian , C. Ren , J. Xia , T. Chen , Z. Yu , and C. Hu , “DiscOvery, Structural Characterization and Functional Analysis of Alpha‐2 Macroglobulin, a Novel Immune‐Related Molecule From *Holothuria atra* ,” Gene 585, no. 2 (2016): 205–215, 10.1016/j.gene.2016. 27033585

[25] Q. Ma , M. Meng , X. Zhou , et al., “Identification of Key Genes in Fetal Gut Development at Single‐Cell Level by Exploiting Machine Learning Techniques,” Proteomics 24, no. 23–24 (2024): e202400104. 39324223 10.1002/pmic.202400104

[26] C. A. de Wit and B. R. Weström , “Purification and CharActerization of α2‐, α2‐β‐ and β‐Macroglobulin Inhibitors in the Hedgehog, *Erinaceus europaeus*: Β‐Macroglobulin Identified as the Plasma Antihemorrhagic Factor,” Toxicon 25 (1987): 1209–1219. 3124298 10.1016/0041-0101(87)90139-5

[27] T. Escalante , A. Rucavado , A. S. Kamiguti , R. D. G. Theakston , and J. M. Gutiérrez , “ *Bothrops asper* Metalloproteinase BaP1 Is Inhibited by α2‐Macroglobulin and Mouse Serum and Does Not Induce Systemic Hemorrhage or Coagulopathy,” Toxicon 43 (2004): 213–217. 15019481 10.1016/j.toxicon.2003.11.012

[28] A. Ochoa , A. T. Hassinger , M. L. Holding , and H. L. Gibbs , “Genetic Characterization of Potential Venom Resistance Proteins in California Ground Squirrels (*Otospermophilus beecheyi*) Using Transcriptome Analyses,” Journal of Experimental Zoology Part B: Molecular and Developmental Evolution 340, no. 3 (2023): 259–269. 35611404 10.1002/jez.b.23145

[29] M. Pomorska‐Mól and K. Kwit , “Markowska‐Daniel I. Major Acute Phase Proteins in Pig Serum from Birth to Slaughter,” Bulletin of the Veterinary Institute in Puławy 56 (2012): 553–557.

[30] A. di Masi , G. de Simone , C. Ciaccio , S. D'Orso , M. Coletta , and A Scenzi P. Haptoglobin , “From Hemoglobin Scavenging to Human Health,” Molecular Aspects of Medicine 73 (2020): 100851. 32660714 10.1016/j.mam.2020.100851

[31] M. J. Oh , S. H. Lee , U. Kim , and H. J. An , “In‐Depth Investigation of Altered Glycosylation in Human Haptoglobin Associated Cancer by Mass Spectrometry,” Mass Spectrometry Reviews 42, no. 2 (2023): 496–518. 34037272 10.1002/mas.21707

[32] O. T. W. Ong , J. M. Green‐Barber , A. Kanuri , L. J. Young , and J. M. Old , “Antimicrobial Activity of Red‐Tailed Phascogale (*Phascogale calura*) Serum,” Comparative Immunology, Microbiology and Infectious Diseases 51 (2017): 41–48. 28504094 10.1016/j.cimid.2017.03.001

[33] M. R. Langlois and J. R. Delanghe , “Biological and Clinical Significance of Haptoglobin Polymorphism in Humans,” Clinical Chemistry 42, no. 10 (1996): 1589–1600. 8855140

[34] S. M. Sadrzadeh and J. Bozorgmehr , “Haptoglobin Phenotypes in Health and Disorders,” American Journal of Clinical Pathology 121 (2004): 97–104. 10.1309/8GLX5798Y5XHQ0VW 15298155

[35] J. Šimunović , J. Gašperšič , U. Černigoj , et al., “High‐Throughput Immunoaffinity Enrichment and N‐Glycan Analysis of Human Plasma Haptoglobin,” Biotechnology and Bioengineering 120, no. 2 (2023): 491–502. 36324280 10.1002/bit.28280

[36] S. Kahl and T. H. Elsasser , “Exogenous Testosterone Modulates Tumor Necrosis Factor‐α and Acute Phase Protein Responses to Repeated Endotoxin Challenge in Steers,” Domestic Animal Endocrinology 31 (2006): 301–311. 16386401 10.1016/j.domaniend.2005.11.005

[37] A. G. Bosanquet , A. M. Chandler , and A. H. Gordon , “Effects of Injury on the Concentration of α1 and α2 in the Plasmas of Male and Female Rats,” Experientia 32 (1976): 1348–1349. 61894 10.1007/BF01953133

[38] J. E. Coe and M. J. Ross , “Hamster Female Protein. A Divergent Acute Phase Protein in Male and Female Syrian Hamsters,” Journal of Experimental Medicine 157 (1983): 1421–1433. 6189935 10.1084/jem.157.5.1421 PMC2187004

[39] B. Ø. Christoffersen , S. J. Jensen , T. P. Ludvigsen , S. K. Nilsson , A. B. Grossi , and P. M. H. Heegaard , “Age‐ and Sex‐Associated Effects on Acute‐Phase Proteins in Göttingen Minipigs,” Comparative Medicine 65 (2015): 333–341. 26310463 PMC4549679

[40] A. J. Zollinger and M. L. Smith , “Fibronectin, the Extracellular Glue,” Matrix Biology 60 (2017): 27–37. 27496349 10.1016/j.matbio.2016.07.011

[41] D. E. Ingber , “Fibronectin Controls Capillary Endothelial Cell Growth by Modulating Cell Shape,” Proceedings National Academy of Science USA 87 (1990): 3579–3583. 10.1073/pnas.87.9.3579 PMC53945 2333303

[42] A. Mackiewicz , I. Kushner , and H. Baumann , Acute Phase Proteins Molecular Biology, Biochemistry, and Clinical Applications (CRC Press, 1993).

[43] T. Hagiwara , H. Suzuki , I. Kono , H. Kashiwagi , Y. Akiyama , and K. Onozaki , “Regulation of Fibronectin Synthesis by Interleukin‐1 and Interleukin‐6 in Rat Hepatocytes,” American Journal of Pathology 136 (1990): 39–47. 2404418 PMC1877464

[44] E. Corradini , E. Buzzetti , P. Dongiovanni , et al., “Ceruloplasmin Gene Variants Are Associated With Hyperferritinemia and Increased Liver Iron in Patients With NAFLD,” Journal of Hepatology 75, no. 3 (2021): 506–513, 10.1016/j.jhep.2021.03.014. 33774058

[45] D. Yang , J. Xiao , M. Wan , J. Liu , L. Huang , and X. Li , “Roxadustat Induces Hepatotoxicity in Zebrafish Embryos via Inhibiting Notch Signaling,” Journal of Applied Toxicology 43, no. 7 (2023): 1073–1082. 36755374 10.1002/jat.4444

[46] K. W. Thomas , S. McOrist , R. E. Hudson , and C. J. McCaughan , “Copper Metabolism in the Koala (*Phascolarctos cinereus*) as Indicated by a Ceruloplasmin Stimulation Test,” Physiological Zoology 59 (1986): 29–34.

[47] S. K. Maciver and P. J. Hussey , “The ADF/Cofilin Family: Actin‐Remodeling Proteins,” Genome Biology 3, no. 5 (2002): reviews3007.1–reviews3007.12, 10.1186/gb-2002-3-5-reviews3007. PMC139363 12049672

[48] D. R. Kovar and C. J. Staiger , “Actin Depolymerizing Factor,” in Actin Depolymerizing Factor, ed. C. J. Staiger , F. Baluška , D. Volkmann , and P. W. Barlow (Dordrecht: Springer Netherlands, 2000), 67–85.

[49] S. Pathak , S. Tripathi , R. Gauba , S. C. Dantu , and A. Kale , “Actin Polymerization: A Cellular Perspective for Motility,” in Actin Polymerization in Apicomplexan: A Structural, Functional and Evolutionary Analysis (Springer, 2019), 1–14.

[50] S. Haase , D. Zimmermann , M. A. Olshina , et al., “Disassembly Activity of Actin‐Depolymerizing Factor (ADF) Is Associated With Distinct Cellular Processes in Apicomplexan Parasites,” Molecular Biology of the Cell 26 (2015): 3001–3012. 26157165 10.1091/mbc.E14-10-1427 PMC4551315

[51] V. A. Bastos , F. Gomes‐Neto , J. Perales , A. G. C. Neves‐Ferreira , and R. H. Valente , “Natural Inhibitors of Snake Venom Metalloendopeptidases: History and Current Challenges,” Toxins 8 (2016): 250. 27571103 10.3390/toxins8090250 PMC5037476

[52] S. L. G. Rocha , B. Lomonte , A. G. C. Neves‐Ferreira , et al., “Functional Analysis of DM64, an Antimyotoxic Protein With Immunoglobulin‐Like Structure from *Didelphis marsupialis* Serum,” European Journal of Biochemistry 269 (2002): 6052–6062. 12473101 10.1046/j.1432-1033.2002.03308.x

[53] S. Lizano , G. Domont , and J. Perales , “Natural Phospholipase A2 Myotoxin Inhibitor Proteins From Snakes, Mammals and Plants,” Toxicon 42 (2003): 963–977. 15019494 10.1016/j.toxicon.2003.11.007

[54] D. H. Drabeck , A. Rucavado , E. Hingst‐Zaher , Y. P. Cruz , A. M. Dean , and S. A. Jansa , “Resistance of South American Opossums to vWF‐Binding Venom C‐Type Lectins,” Toxicon 178 (2020): 92–99. 32135198 10.1016/j.toxicon.2020.02.024 PMC8522506

[55] R. M. Werner , L. M. Miling , B. M. Elliott , M. R. Hawes , J. M. Wickens , and D. E. Webber , “Bacterial Expression of a Snake Venom Metalloproteinase Inhibitory Protein From the North American Opossum (*D. virginiana*),” Toxicon 194 (2021): 1–10. 33581173 10.1016/j.toxicon.2021.01.008

[56] S. L. Rocha , A. G. Neves‐Ferreira , M. R. Trugilho , et al., “Crotalid Snake Venom Subproteomes Unraveled by the Antiophidic Protein DM43,” Journal of Proteome Research 8, no. 5 (2009): 2351–2360. 19267469 10.1021/pr800977s

[57] D. H. Drabeck , A. Rucavado , E. Hingst‐Zaher , A. Dean , and S. A. Jansa , “Ancestrally Reconstructed von Willebrand Factor Reveals Evidence for Trench Warfare Coevolution Between Opossums and Pit Vipers,” Molecular Biology and Evolution 39, no. 7 (2022): msac140. 35723968 10.1093/molbev/msac140 PMC9255381

[58] C. A. Whittaker and R. O. Hynes , “Distribution and Evolution of Von Willebrand/Integrin A Domains: Widely Dispersed Domains With Roles in Cell Adhesion and Elsewhere,” Molecular Biology of the Cell 13 (2002): 3369–3387. 12388743 10.1091/mbc.E02-05-0259 PMC129952

[59] D. Tuckwell , “Evolution of Von Willebrand Factor A (VWA) Domains,” Biochemical Society Transactions 27 (1999): 835–840. 10830113 10.1042/bst0270835

[60] X. Zhan , J. He , L. Yu , et al., “Identification of a Novel Thrombospondin‐Related Anonymous Protein (BoTRAP2) From *Babesia orientalis* ,” Parasit Vectors 12 (2019): 200. 31053087 10.1186/s13071-019-3457-0 PMC6500065

[61] S. J. Perkins , K. F. Smith , S. C. Williams , P. I. Haris , D. Chapman , and R. B. Sim , “The Secondary Structure of the Von Willebrand Factor Type A Domain in Factor B of Human Complement by Fourier Transform Infrared Spectroscopy: Its Occurrence in Collagen Types VI, VII, XII and XIV, the Integrins and Other Proteins by Averaged Structure Predictions,” Journal of Molecular Biology 238 (1994): 104–119. 8145250 10.1006/jmbi.1994.1271

